# Comparison of standard circular stapler anastomosis with or without circumferential suture enhancement in patients with robot-assisted Ivor-Lewis oesophagectomy due to malignant tumours of the oesophagus and oesophagogastric junction—a multi-centre, randomised, superiority study (STITCHES)

**DOI:** 10.1186/s13063-025-08995-4

**Published:** 2025-09-16

**Authors:** Janik Hinkelmann, Helena Reitberger, Bruno Bancke Laverde, Andreas R. R. Weiß, Maximilian Brunner, Robert Grützmann, Georg F. Weber, Christian Krautz

**Affiliations:** Department of General and Visceral Surgery, Friedrich-Alexander-Universität Erlangen-Nürnberg, Krankenhausstraße 12, Erlangen, Germany

**Keywords:** RAMIE, Oesophagectomy, Oesophageal cancer, Oesophagogastric anastomosis, Suture reinforcement, Anastomotic leak

## Abstract

**Background:**

Morbidity due to anastomotic leakage is a major concern in transthoracic oesophagectomy. The aim of this randomised trial is to evaluate whether a circumferential suture reinforcement of the stapled end-to-side anastomosis in robot-assisted minimally invasive Ivor-Lewis oesophagectomy (RAMIE) leads to a reduced incidence of anastomotic leakages in the postoperative course.

**Methods/design:**

This is a multi-centre randomised, double-blind, superiority trial with an adaptive sample size design undergoing RAMIE for malignant tumours. Patients will be randomised 1:1 into two study arms. In study arm A, participants will receive a standard circular-stapled end-to-side oesophagogastric anastomosis, while in study arm B, the anastomosis will have a circumferential suture reinforcement. The primary endpoint is the rate of anastomotic leakage. Secondary endpoints are incision-to-suture time, duration of circumferential suture reinforcement, anastomotic stenosis rate, postoperative morbidity and mortality, and quality of life.

**Discussion:**

This randomised controlled trial will assess the impact of circumferential suture reinforcement of the oesophagogastric anastomosis on short-term outcomes and quality of life of patients undergoing robot-assisted minimally invasive Ivor-Lewis oesophagectomy.

**Trial regsitration:**

DRKS00034787. Registered on 7 October 2024.

**Supplementary Information:**

The online version contains supplementary material available at 10.1186/s13063-025-08995-4.

## Background

In the modern multimodality treatment of oesophageal cancer, oesophagectomy has a fundamental role. However, due to its complexity and invasiveness, it is defined as a high-risk procedure that is associated with a higher morbidity than most other surgical procedures. In Germany, a population-based observational study using administrative data found a nationwide caseload-related in-hospital mortality rate ranging between 12.3% (95% confidence interval: 11.1–13.7) and 20.0% (18.5–21.6) [13]. Recent studies have shown a significant improvement in perioperative outcomes with minimally invasive approaches [11]. As a result, the German guideline for oesophageal cancer from the German Guideline Program in Oncology (GGPO) recommends the use of minimally invasive oesophagectomy (MIE) [5]. Robot-assisted minimally invasive oesophagectomy (RAMIE) is a technical advancement that may lead to further improvements in patient outcomes. Compared with MIE, RAMIE can achieve shorter operative times and better lymph node yield in patients who have received neoadjuvant therapy [18].

Irrespective of the type of surgical approach, the formation of the oesophagogastric anastomosis is critical to the success of the operation, as an anastomotic leakage is associated with significant morbidity and mortality [13, 15]. In Ivor-Lewis RAMIE, three distinct anastomotic techniques are currently in use [15]. The anastomosis can be sutured by hand, stapled in a linear fashion side to side, or in a circular fashion end to side. A recent analysis of a multi-centre international registry on RAMIE revealed that the circular-stapled anastomotic technique is employed in 52% of cases, in comparison to 30% for hand-sewn and 18% for linear stapled anastomotic techniques [8]. The highest anastomotic leakage rate was observed in the robot-assisted hand-sewing group (33%), while lower rates were noted in the circular stapling (17%) and linear stapling (15%) groups. These relatively high rates are in contrast with the average incidence of anastomotic leakage of 6.2% reported in a meta-analysis comparing RAMIE with open oesophagectomy [6].

In order to reduce the incidence of anastomotic leakage, different techniques of anastomotic reinforcement have been proposed in the past, including the use of tissue sealant, omental flap and additional suture reinforcement [2, 16, 17].

In Ivor-Lewis RAMIE, the additional use of sutures for reinforcement has been proposed as a crucial measure to reduce leakage rates in end-to-side oesophagogastrostomy performed with the conventional circular stapler technique [6]. However, no studies have yet investigated the potential advantages of supplementary suture reinforcement in terms of anastomotic healing outcomes in robot-assisted Ivor-Lewis oesophagectomy.

Given the above, we aim to investigate whether a circumferential suture reinforcement of the circular-stapled anastomosis (study arm B) is superior to the standard anastomosis without reinforcement (study arm A). The primary endpoint is the rate of anastomotic leakage. Secondary endpoints include incision-to-suture time, duration of circumferential suture reinforcement, anastomotic stenosis rate, postoperative morbidity and mortality, and quality of life.

## Methods

### Aim of the study

This study aims to investigate whether a circumferential suture reinforcement of the standard circular-stapled end-to-side oesophagogastric anastomosis in patients undergoing Ivor-Lewis RAMIE for oesophageal cancer can lower the incidence of anastomotic leakages and thereby reduce postoperative morbidity and mortality. The primary objective of the study is to evaluate the potential for a significant reduction in the rate of anastomotic leakage. It is hypothesised that this will influence secondary outcomes, including postoperative morbidity, mortality and quality of life, but not the rate of anastomotic stenosis.

### Trial design, study registration and ethics

The STITCHES trial is designed as a randomised controlled multi-centric, two-arm parallel-group superiority surgical trial with a control group (study arm A: RAMIE with circular-stapled end-to-side oesophagogastric anastomosis without circumferential suture reinforcement) and an interventional group (study arm B: RAMIE with circular-stapled end-to-side oesophagogastric anastomosis with circumferential suture reinforcement).

The study is coordinated by the leading study centre of the Department of Surgery, University Hospital Erlangen, Germany. The study was initiated at the Department of Surgery, University Hospital Erlangen. It is anticipated that additional centres will be evaluated for participation, if it can be demonstrated that equivalent surgical standards (RAMIE procedure with equal technique) and trial management can be guaranteed at these sites. This trial was registered in advance at the German Clinical Trials Register (DRKS00034787, https://drks.de/search/en/trial/DRKS00034787) on 7 October 2024.

The study protocol was approved by the Ethical Committee at the Friedrich-Alexander-University Erlangen-Nuremberg (24–205-B). Any modifications to the protocol will require a formal amendment. Proposed amendments will be subject to further evaluation and approval. The principal investigator will notify participating centres and will provide a copy of the revised protocol to the responsible investigator. An update of the trial register will be initiated.

The trial will be conducted in line with the Declaration of Helsinki in its current version and with the laws and declaration of the concerned country. In accordance with the Medical Association’s Professional Code of Conduct, the Ärztliche Berufsordnung of each participating German state, an independent Ethics Committee (IEC) must be consulted prior to the commencement of the clinical study in order to address any questions pertaining to professional ethics and legal issues associated with the study. To this end, the study protocol, patient information sheet and informed consent must be submitted to the responsible Ethics Committee.

**Fig. 1:**
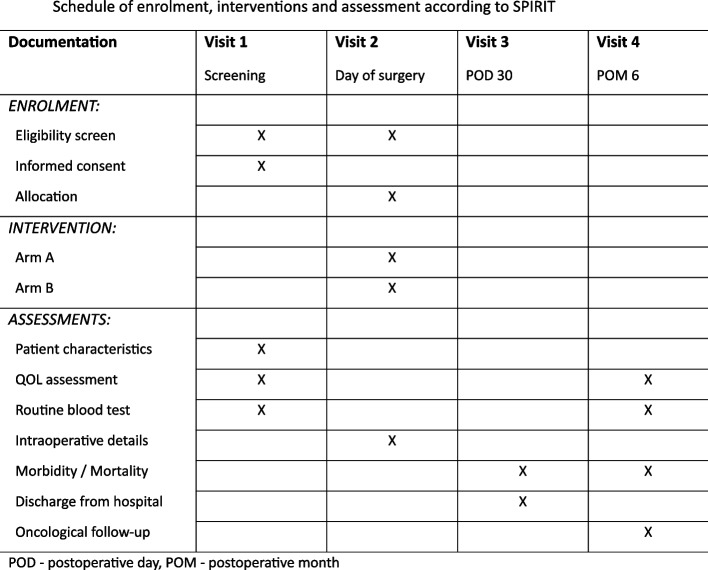
Schedule of enrolment, interventions and assessment according to SPIRIT

The study is designed according to the SPIRIT 2013 statement [4]. The SPIRIT checklist was applied and a SPIRIT figure (Fig. 1) was generated.

### Eligibility

To be able to participate in this study, patients must fulfil several inclusion criteria and be checked for exclusion criteria. These are listed below.

#### Inclusion criteria

All patients scheduled for elective Ivor-Lewis RAMIE for malignant tumours (adenocarcinoma and squamous cell carcinoma) of the oesophagus and oesophagogastric junction are eligible for inclusion. Further inclusion criteria are as follows: intrathoracic circular-stapled end-to-side oesophagogastrostomy, oesophageal carcinoma and carcinomas of the gastrooesophageal junction (Siewert type 1 and 2), age ≥ 18 years.

#### Exclusion criteria

Patients who are not capable of providing informed consent, pregnant or breastfeeding individuals, conversion to open surgery and malfunction of the stapler.

#### Withdrawal criteria

Patients may withdraw from the trial at any time without providing justification. Exclusion from the trial will occur in cases where RAMIE is not performed, for instance, due to technical irresectability, conversion to conventional resection or metastatic disease. However, the withdrawal and reasons will be documented in the patient file and the CRF (case report form), and a CONSORT flow diagram will be provided to ensure comprehensive transparency.

### Interventions

#### Surgical technique

To preclude the potential for learning-associated bias, surgeons with the requisite proficiency will be permitted to perform the randomised interventions. Surgeons participating in the trial as the responsible surgeons must have a minimum of 20 RAMIEs of personal operative experience. This threshold is based on the lower variance range of the learning curves for RAMIE, as determined in a recent systematic review [14]. It should be noted that the standard technique and surgical instruments may vary in several aspects. Nevertheless, the following guidelines have been defined for the present study to reduce the impact of performance bias:

- The abdominal and thoracic parts of the procedure are performed robotically.
- A circular, end-to-side oesophagogastrostomy is performed after gastric tube pull-up via a muscle-sparing mini-thoracotomy.
- A circular stapler with a size range of 25–29 mm is employed.
- The gastric conduit stump is closed with a linear stapler, with the shortest distance between the linear and circular stapler lines being at least 1 cm.
- Randomisation is conducted intraoperatively, after the completion of the oesophagogastrostomy.
- The circumferential suture reinforcement (study arm B) may be performed in an interrupted or continuous fashion with resorbable material (e.g. PDS 4–0, V-loc 4–0). Any type of surgical stitching technique or suture pattern is allowed, provided that the entire circular stapler line is addressed. In case of interrupted suture technique, the number of sutures required is dependent upon the size of the anastomosis.
- No additional measures to reinforce the anastomosis (e.g. omental flap, pleural flap) are permitted.
- The placement of drains is at the surgeon’s discretion.
- Patients do not receive a gastric tube.
- Perioperative complications associated with oesophagectomy are treated according to current best practices and clinical standards.

### Assignment and randomisation

Patients who have been screened and deemed eligible will be included in the trial following the provision of written informed consent. Randomisation will be conducted intraoperatively, after the completion of the oesophagogastrostomy and verification of the exclusion criteria. The randomisation lists were generated by block randomisation (block size 10) by a computer-based random number generator using the R statistical package (version 4.4.1). The lists were incorporated into the REDCap randomisation tool. The randomisation process will be conducted by authorised trial personnel using the electronic REDCap system.

### Objectives

#### Primary endpoint

The primary endpoint of this study is defined as anastomotic leakage (any type), as described by the Esophagectomy Complications Consensus Group [10]. A separated and pooled analysis (type 1, type 2, type 3 and types 1, 2 and 3) will be conducted.

The primary hypothesis is that the circumferential suture reinforcement of the oesophagogastrostomy will result in a reduction in the rate of anastomotic leakage in Ivor-Lewis RAMIE patients.

#### Secondary endpoints

Secondary endpoints include incision-to-suture time, duration of circumferential suture reinforcement, anastomotic stenosis rate, postoperative morbidity and mortality according to the Clavien–Dindo classification (CDC), and quality of life. Perioperative complications associated with oesophagectomy are recorded in accordance with the ECCG definitions.

Postoperative morbidity with CDC grade ≥ 3b will be the secondary safety endpoint. Quality of life (QoL) is assessed using the EORTC QLQ-C30, EORTC QLQ-OES18 and EORTC QLQ-OG25 questionnaires [1, 3, 9], provided by the European Organisation for Research and Treatment of Cancer.

### Trial visits

A total of four trial visits will be conducted throughout the course of the trial. During the initial screening visit (visit 1), inclusion criteria and two out of four exclusion criteria will be evaluated. Once the patient has provided informed consent, the following patient characteristics are documented: basic demographic data, comorbidities, the modified frailty index (mFI), American Society of Anesthesiologists (ASA) and Eastern Cooperative Oncology Group (ECOG) scores, as well as tumour-specific parameters. Furthermore, patients are required to complete questionnaires assessing their preoperative quality of life. Quality of life will be evaluated using the aforementioned EORTC questionnaires, depending on the tumour localisation.

The second visit (visit 2) is scheduled for the day of surgery. Once the oesophagogastrostomy has been completed and none of the remaining two exclusion criteria (conversion and malfunction of the stapler) applies, the patient will be randomised using the REDCap randomisation tool. Intraoperative parameters will be collected, including incision-to-suture time, additional suture time for the anastomosis (study arm B), characteristics of the used stapler and intraoperative transfusions of packed red blood cells. Furthermore, the surgeon’s subjective intuition (subjective risk assessment) regarding four postoperative complications (anastomotic leak, conduit necrosis, chyle leak and recurrent laryngeal nerve injury/palsy) will be assessed.

The third and fourth study visits (visit 3 = 30 days postoperative and visit 4 = 6 months postoperative) may be conducted either as part of standard follow-up visits or by telephone. Visit 4 is considered to be part of the standard oncological follow-up. This encompasses a CT scan of the thorax and abdomen, in addition to an esophagogastroduodenoscopy, should clinical symptoms of dysphagia be observed. The primary and secondary endpoints, along with any serious adverse events (SAEs and AEs), will be evaluated and documented during each visit on the case report form (CRF). Data from patients with stapler malfunction, conversion to open surgery or inoperability will be documented until the time of the operation (visit 2). During visits 1 and 4, the standard blood tests (including haemoglobin concentration, leucocyte count and serum C-reactive protein) will be carried out and documented.

### Assessment of safety

Any adverse events (AEs) and serious adverse events (SAEs) that occur during the course of the study will be documented. AE is defined as any event that deviates from the norm of the perioperative course of a robot-assisted oesophagectomy. SAE is defined as any event that can occur at any time during the study and either leads to death, threatens the patient’s life, results in persistent or significant impairment or disability of the patient. SAEs must be reported to the principal investigator within 24 h. The principal investigator is responsible for notifying the responsible ethics committee, if SAEs occur.

Automated blinded reports of the REDCap online database platform, including the secondary safety endpoint (CDC grade ≥ 3b), will be available to the steering committee on a continuous basis. These reports will be subject to monthly review and help to detect any unexpected increase in postoperative risk associated with the intervention. In case of imbalances between groups, additional safety review may be initiated. The study may be terminated prematurely by the steering committee or the responsible ethics committee in the following cases: in the event of medical or ethical concerns that preclude the continuation of the study (e.g. secondary safety endpoint or SAEs indicating a potential health risk associated with study participation), inadequate patient recruitment or the emergence of external evidence suggesting the termination of the study.

### Statistics

#### Statistical considerations

All analyses will be performed on an intention-to-treat basis, which comprises all patients randomised with successful RAMIE. Discrete endpoints including the primary endpoint will be described with the use of frequencies and percentages with 95% confidence intervals and will be compared by the chi-square test or Fisher’s exact test. Although randomisation is expected to balance baseline characteristics, additional multivariate analysis will be performed for variables affecting binomial endpoints to control for potential residual confounding. This analysis will use a multivariable logistic regression model including clinically relevant covariates that may influence anastomotic healing. These variables include, but are not limited to age, body mass index (BMI), tumour histology, neoadjuvant treatment and stapler diameter. Adjusted odds ratios (ORs) with 95% confidence intervals will be reported.

Continuous outcomes such as incision-to-suture time will be compared between groups using either Student’s *t*-test (if normally distributed) or the Mann–Whitney *U* test (if skewed). Ordinal outcomes, such as the Clavien–Dindo complication grading, will be analysed with non-parametric tests like the Mann–Whitney *U* test.

Quality-of-life scores obtained from the EORTC QLQ-C30, QLQ-OES18 and QLQ-OG25 questionnaires at baseline and 6 months postoperatively will be assessed using repeated measures analysis, such as mixed-effects linear models, to account for within-subject correlation over time. For any time-to-event outcomes (e.g. time to readmission or death), Kaplan–Meier survival analysis and the log-rank test will be applied, and, if appropriate, a Cox proportional hazards model will be used to estimate hazard ratios with 95% confidence intervals.

All statistical tests will be two-sided, with a significance level set at *α* = 0.05. Sensitivity analyses will be performed using the per-protocol population. All analyses will be fully specified in a statistical analysis plan that is written prior to database closure.

#### Sample size calculation

Despite an extensive literature search, no studies could be found comparing oesophagogastric anastomosis using a circular-stapled, end-to-side technique with circumferential suture reinforcement in a minimally invasive setting. Due to the lack of reliable data to calculate the sample size at the time of study preparation, an adaptive sample size design was chosen. A total of 100 patients (50 per arm) will be enrolled initially, with the option to adjust the sample size, if necessary. After enrolment of the first 100 patients, an interim analysis is planned to determine the final sample size. The criteria for the continuation of the study are an absolute difference in the primary endpoint of at least 1% to justify the clinical relevance of the study and a calculated sample size of a maximum of 1000 patients per arm to ensure the feasibility of the study.

Although robust prospective data comparing circular-stapled oesophagogastric anastomosis with and without circumferential suture reinforcement in RAMIE are lacking, a preliminary power calculation was performed based on current literature to justify the adaptive design and interim analysis. International multi-centre registry data on RAMIE procedures report an anastomotic leakage rate of approximately 17% for circular-stapled anastomoses without additional suture reinforcement [8]. In contrast, a large retrospective propensity score-matched analysis by Tu et al. [17] demonstrated a significant reduction in leakage rates from 10.3% to 4.7% following circumferential suture reinforcement in circular-stapled anastomoses during McKeown oesophagectomy. Based on these findings, we conservatively assume a reduction from 17 to 11% as clinically meaningful in our study population.

Using these assumptions, a two-group chi-squared test with a significance level of 0.05 (two-sided) and a power of 80% yields a required sample size of approximately 525 patients per arm to detect a difference of 6 percentage points. This supports the current design’s feasibility boundary of up to 1000 patients per arm and justifies continuation after interim analysis if the observed difference in leakage rates exceeds 1%. Moreover, in case of early evidence for superiority (i.e. absolute difference ≥ 5% with *p* < 0.01 in the primary endpoint), the study may be terminated early upon recommendation by the steering committee.

These criteria and calculations will be included in the formal statistical analysis plan prior to the interim analysis.

#### Blinding

The STITCHES trial is a double-blind study. As it is not feasible to blind the surgeon, the patients and the follow-up investigators are blinded. The case report forms, which can be viewed by the follow-up investigators, do not specify which study arm the patients belong to. To ensure impartiality, the postoperative study visits are not carried out by the surgeon.

### Data processing

#### Patient education and written informed consent

All patients who meet the eligibility criteria for participation in the STITCHES study are required to provide informed consent. The objectives, risks and benefits of the study will be outlined to patients by authorised investigators. Furthermore, patients will be provided with written patient information. In order to become study participants, patients are required to sign the declaration of consent. Patients who have consented to participate in the study may withdraw his/her consent at any time without providing an explanation. If a patient withdraws their consent and demands data deletion, the data collected up to that point will be deleted.

#### Data collection

To achieve study results that are comparable to those of other trials regarding oesophagectomy, the assessment of postoperative morbidity in the STITCHES trial is based on the recommendations of the Esophagectomy Complication Consensus Group (ECCG) [8]. During each study visit, all parameters of interest, including primary and secondary endpoints and AEs/SAEs, are evaluated and documented in accordance with the standardised eCRF.

Visit 1: The following patient characteristics are documented: basic demographic data (age, sex, height, weight and BMI), mFI, ASA and ECOG scores, as well as tumour-specific parameters (tumour type and location, TNM classification, and the prevalence and type of neoadjuvant treatment). A preoperative blood test is conducted including serum albumin, creatinine, haemoglobin, leukocytes, CRP, vitamin B12, vitamin D3 and tumour markers CA 19–9 and CEA. Furthermore, each patient is required to complete the EORTC QoL questionnaires.

Visit 2: On the day of surgery, the incision-to-suture time, duration of additional suture of the anastomosis (if randomised to study arm B), type of stapler (manufacturer, diameter, powered stapler, yes or no) and the number of intraoperative blood transfusions are recorded.

To document the duration of suture reinforcement of the stapled anastomosis, the timer is started with the initial stitch and stopped with the cutting of the suture. Furthermore, a subjective risk assessment is conducted by the surgeon regarding four postoperative complications: anastomotic leak, conduit necrosis, chyle leak and recurrent laryngeal nerve injury/palsy.

Visit 3: This study visit encompasses possible postoperative mortality and morbidity, the necessity for blood transfusions, the date of discharge and the occurrence of readmission to the hospital. The discharge information comprises the location of discharge, whether at home or to a further medical facility. Morbidity is evaluated and documented in accordance with the ECCG criteria and graded according to Clavien–Dindo.

Visit 4: This study visit includes all parameters of visit 3. Furthermore, an oncological follow-up (suspected and/or confirmed relapse), as well as a routine blood test and an assessment of quality-of-life following surgery (corresponding to visit 1) are required.

#### Documentation

It is the responsibility of the investigator to ensure that the study is conducted in accordance with the professional code for physicians, the Declaration of Helsinki in its current version and the study protocol. A sound documentation of the data is mandatory. All data collected in this study must be entered into the eCRF by appropriately authorised persons. This also applies to data from persons who have been excluded from the study. The study site records participation on a patient identification list. This list is utilised for the identification of participating individuals and comprises the patient number, full name, date of birth and date of admission to the STITCHES trial. The patient identification list remains at the trial centre after the study is completed.

The audit trial functionality within the REDCap online database automatically logs all data and associated corrections with the date, time and the individual responsible for the entry. This enables the retrieval of previous entries at any time. Records and documents related to the STITCHES trial must be retained by the investigator for 10 years in accordance with Good Clinical Practice.

### Trial sponsor, data management and quality assurance

The Centre for Clinical Studies of the Department of Surgery at the University Hospital Erlangen is the sponsor of this investigator-initiated trial. The principal investigator, who assumes medical responsibility, and their team provide support for the trial.

The data management and documentation are conducted via the REDCap system. Only those with the requisite authorisation are permitted to enter data into the eCRF on REDCap. All patient-related information is subject to medical confidentiality according to the European General Data Protection Regulation (Datenschutzgrundverordnung), the Federal Data Protection Act (Bundesdatenschutzgesetz) and the State Data Protection Act (Landesdatenschutzgesetz). Third parties will not have insight into the original data.

The data monitoring is conducted by the Centre for Clinical Studies of the Department of Surgery at the University Hospital Erlangen in accordance with the pertinent data protection regulations. The Centre for Clinical Studies of the Department of Surgery at the University Hospital Erlangen and the principal investigator are designated as the primary contact for addressing any uncertainties regarding the collected data. The data are subjected to range, validity and consistency checks. Any missing data must be explained by a statement. The site staff is responsible for data correction and will facilitate the resolution of data discrepancies. On-site audits are not planned.

## Discussion

Anastomotic leakage represents one of the most severe postoperative complications, with a relatively high incidence of 14.2%, as recently reported from a large international multi-centre cohort study involving 2247 oesophagectomies [7]. A nationwide cohort study from Germany has shown that this complication can be associated with a significantly increased hospital mortality rate of up to 28% [13]. It is therefore crucial to identify strategies that can reduce the incidence of anastomotic leaks.

In comparison to other digestive tract reconstructions, oesophagogastrostomy is frequently more challenging to heal, which may be attributed to the absence of an oesophageal serosal layer and the fact that the external longitudinal muscle fibres are barely able to hold the suture material. In considering this, different authors have postulated that higher tension on the anastomosis was more likely to result in anastomotic leakage, thereby emphasising the necessity for reinforcement of the anastomosis [12, 17].

Previously, various techniques for anastomotic reinforcement have been proposed with the aim to reduce leakage rates. Tu et al. reported in a retrospective propensity score-matched analysis that a circumferential suture reinforcement of the circular-stapled anastomosis in McKeown oesophagectomy significantly reduced leakage from 10.3% to 4.7% [17]. In this study, interrupted horizontal mattress sutures were employed to wrap the stapler line. The authors stated that the stitches were positioned in a manner that was perpendicular to the outer layer of the oesophageal muscle fibres, thereby effectively reducing tension on the anastomosis and preventing tissue tearing [17].

A review of the literature reveals a paucity of randomised controlled trials investigating the potential advantages of circumferential suture reinforcement of circular-stapled oesophagogastrostomy in patients undergoing RAMIE for oesophageal cancer. In particular, there are no studies examining this approach in Ivor-Lewis RAMIE. This randomised controlled multi-centre trial was therefore initiated to compare the standard circular-stapled oesophagogastrostomy with the suture-reinforced circular-stapled oesophagogastrostomy in Ivor-Lewis RAMIE regarding postoperative complications. The primary objective of the study is to investigate whether the incidence of anastomotic leakage can be significantly reduced using circumferential suture reinforcement. It is anticipated that this will have an impact on secondary endpoints, including postoperative morbidity and mortality, anastomotic stenosis rate, as well as quality of life. In addition, the additional time required and the surgeon’s subjective intuition regarding postoperative complications will be assessed.

## Trial status

### Recruiting

The first patient was enrolled on 28 November 2024. It is anticipated that recruitment will finish in October 2026. Current study protocol version 2.0; 15.01.2025.

## Supplementary Information

Additional file 1: SPIRIT checklist.

## Data Availability

The informed consent and the patient information materials are available on request from the author.

## Abbreviations

CCI: Charlson Comorbidity Index
CDC: Clavien–Dindo classification
mFI: Modified frailty index
ASA: American Society of Anesthesiologists
ECOG: Eastern Cooperative Oncology Group
EORTC: European Organisation for Research and Treatment of Cancer
ECCG: Esophagectomy Complications Consensus Group
CRF: Case report form
BMI: Body mass index
QoL: Quality of life
RAMIE: Robot-assisted minimally invasive oesophagectomy
AE: Adverse event
SAE: Serious adverse event
